# Reproducibility and relative validity of a semi-quantitative food frequency questionnaire for Chinese pregnant women

**DOI:** 10.1186/s12937-015-0044-x

**Published:** 2015-06-04

**Authors:** Hongmin Zhang, Xiang Qiu, Chunrong Zhong, Kewei Zhang, Mei Xiao, Nianhua Yi, Guoping Xiong, Jing Wang, Jing Yao, Liping Hao, Sheng Wei, Nianhong Yang, Xuefeng Yang

**Affiliations:** 1 Department of Nutrition and Food Hygiene, Hubei Key Laboratory of Food Nutrition and Safety, MOE Key Laboratory of Environment and Health, School of Public Health, Tongji Medical College, Huazhong University of Science and Technology, 13 Hangkong Road, Wuhan, 430030 Hubei China; 2 Hubei Maternal and Child Health Hospital, Wuhan, 430070 Hubei China; 3 The Central Hospital of Wuhan, Wuhan, 430014 Hubei China; 4 Jiangan Maternal and Child Health Hospital, Wuhan, 430014 Hubei China; 5 Department of Epidemiology and Biostatistics, MOE Key Laboratory of Environment and Health, School of Public Health, Tongji Medical College, Huazhong University of Science and Technology, Wuhan, 430030 Hubei China

**Keywords:** Food frequency questionnaire, Validity, Reproducibility, Pregnant women

## Abstract

**Background:**

Food frequency questionnaire (FFQ) is a reliable tool to estimate dietary intake in large nutritional epidemiological studies, but there is lack of a current and validated FFQ for use in urban Chinese pregnant women. This study aimed to evaluate the reproducibility and validity of a semi-quantitative FFQ designed to estimate dietary intake among urban pregnant women in a cohort study conducted in central China.

**Methods:**

In the reproducibility study, a sample of 123 healthy pregnant women completed the first FFQ at 12–13 weeks gestation and the second FFQ 3–4 weeks later. To validate the FFQ, the pregnant women completed three 24-h recalls (24HRs) between the intervals of two FFQs.

**Results:**

The intraclass correlation coefficients of two administrations of FFQ for foods ranged from 0.23 (nuts) to 0.49 (fruits) and for nutrients from 0.24 (iodine) to 0.58 (selenium) and coefficients were all statistically significant. The unadjusted Pearson correlation coefficients between two methods ranged from 0.28 (beans) to 0.53 (fruits) for foods and from 0.15 (iodine) to 0.59 (protein) for nutrients. Energy-adjusted and de-attenuated correlation coefficients for foods ranged from 0.35 (beans) to 0.56 (fruits) and for nutrients from 0.11 (iodine) to 0.63 (protein), and all correlations being statistically significant except for iodine, sodium and riboflavin. On average, 67.0 % (51.2 %-80.5 %) of women were classified by both methods into the same or adjacent quintiles based on their food intakes, while 68.5 % (56.1 %-77.2 %) of women were classified as such based on nutrient intakes. Extreme misclassifications were very low for both foods (average of 2.0 %) and nutrients (average of 2.2 %). Bland-Altman Plots also showed reasonably acceptable agreement between two methods.

**Conclusion:**

This FFQ is a reasonably reliable and valid tool for assessing most food and nutrient intakes of urban pregnant women in central China.

## Introduction

Optimal maternal nutrition plays an important role in beneficial birth outcomes and the long-term health of both mother and offspring [1, 2]. Overnutrition during pregnancy leads to maternal obesity and excessive gestational weight gain and becomes the prominent nutritional problem for pregnant women in developed countries [3]. Recent investigation from an urban Chinese population reported that 11.9 % of pregnant women were overweight (BMI = 24.0–27.9 kg/m^2^) before pregnancy and 63.9 % of which had excessive weight gain during pregnancy [4]. It poses a significant maternal and fetal risk including an increase incidence of pre-eclampsia, gestational diabetes, macrosomia and risk of some chronic disease in later life of offspring [5–7]. Therefore, appropriate dietary assessment and study on the association between dietary factors and related health outcomes is needed in this population.

The food frequency questionnaire (FFQ) is currently the most frequently used method to estimate dietary intake in large nutritional epidemiological studies [8], because it is relatively easy to use, inexpensive and can better reflect the long-term dietary intake patterns of study populations. FFQ has been found to be a valid and reliable tool for assessing nutrient or food intakes for pregnant women in different countries including China [9–15]. Both Li et al. [14] and Cheng et al. [15] evaluated the reproducibility and validity of FFQ used in pregnant women of China, but they only focused on the population from rural area of China. In the present Tongji Maternal and Child Health Cohort (TMCHC) study, we developed a new semi-quantitative FFQ to estimate the nutrient and food group intakes among urban pregnant women from central China. The TMCHC, initiated in 2013 in Wuhan of China, was a population-based prospective cohort study with major objective to investigate the association between maternal nutrition and health outcomes of mother and offspring. Because dietary habits vary greatly in population with different regional, ethnic or cultural background, the FFQ should be tailored and shown to be reliable and valid for use in a specific population.

Therefore, the aim of present study was to assess the validity and reproducibility of the semi-quantitative FFQ used to evaluate the dietary intake among urban pregnant women from central China.

## Subjects and methods

### Subjects and design

**Fig. 1:**
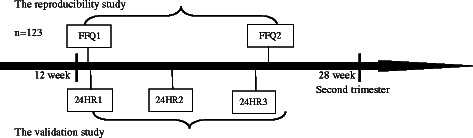
The design of the reproducibility and validation study among 123 pregnant Chinese women, January 2013 to April 2014

Pregnant women at 12–13 weeks of gestation were invited to participate in a cohort study when they attended their first antenatal visit at the maternity clinics in three public hospitals in Wuhan, the largest city in central China. A subsample of 123 participants was enrolled in this study from January 2013 to April 2014 in an ongoing cohort. As shown in Fig. 1, they were required to complete two administrations of FFQs and three 24-h recalls (24HRs) during their second trimesters. In the reproducibility study, the first FFQ was administrated by a trained interviewer at enrollment and the second FFQ at following visit three to four weeks later. To validate the FFQ, three 24-h recalls were completed between the intervals of two FFQs.

Trained interviewers conducted face-to-face interviews at the initial and following visits. In the initial interview, the information on demographic and lifestyle variables during the first trimester was obtained by using a structured questionnaire. Pre-pregnancy body mass index (BMI) was calculated from self-reported pre-pregnancy weight and height measured at the first visit. Women were classified according to the Chinese cutoffs of BMI as being underweight (BMI < 18.5 kg/m^2^), normal weight (BMI = 18.5–23.9 kg/m^2^), overweight (BMI = 24.0–27.9 kg/m^2^) or obese (BMI ≥ 28.0 kg/m^2^) before pregnancy [16]. Educational level was recorded as number of completed schooling years and categorized as ≤9, 10–12, 13–15 and ≥16 years.

This study was approved by the Institutional Review Board of Tongji Medical College of Huazhong University of Science and Technology and had therefore been performed in accordance with the ethical standards laid down in the Declaration of Helsinki. All participants provided written informed consent prior to their inclusion in the study.

### FFQ

The interviewer-administrated semi-quantitative FFQ developed in this study consisted of 61 food items which were assembled into 10 food groups: cereals, meats, fish, eggs, beans, vegetables, fruits, nuts, milk and milk products and beverages. Food items were formed on the basis of food nutrient composition and eating habits of Chinese. For each item, the participants were asked to recall their usual frequency of intake and portion size during the past 4 weeks. Frequency of consumption was reported as one of fifteen categories from “never during the past 4 weeks” to “five or more times per day”. Food models representing standard portion size for most of the food items were prepared to help subjects to estimate their usual consumption. A color food photography atlas with different portion sizes of all food items was used to make the estimation more accurate [17, 18]. Other usual dietary information such as the frequency of eating out, personal choices for cooking oil and preference of taste was also included in the questionnaire. Daily dietary intake was estimated as the intake (in grams) per day according to the frequency and portion size of a specific food item. As this analysis only focused on foods and nutrients obtained from the diet, the data about dietary supplements were collected but not shown in this study.

### 24-h recall

The 24-h recall was chosen as the reference method to validate the FFQ. Participants were asked to complete three un-consecutive 24-h recalls during the intervals of two administrations of FFQ. The three 24-h recalls included two weekdays and one weekend day. At enrollment, the participants were given the instructions on how to complete the 24-h recalls. They were required to recall all foods or beverages consumed in the past 24 h and to estimate the portion size. Food models and pictures of different portion size together with commonly used household measures (bowls and plates of serving size) were used to facilitate the estimation of portion size [17, 18]. Other dietary information including recipe ingredients, cooking methods of dishes, and the time and place (e.g. home or outside home) of their consumption was also collected. A trained nutritionist, who had received professional training and had two years’ experience of dietary survey, checked all records for completeness and accuracy and then coded them.

### Nutrients intake analysis

Primary data obtained from both FFQs and 24-h recalls were double entered into the EpiData software by two trained workers to verify accuracy. The daily food group and nutrient intakes were calculated by a dietary software based on the China Food Composition Database [19, 20], which was continuously updated and included 2263 individual food items and mixed dishes and more than 90 nutrients and other dietary factors. Data of food consumption and nutrient intake exported from the dietary software were imported into Microsoft Excel for statistical analysis.

### Statistical analysis

Statistical analyses were performed by using Empower Stats software, version 2.14.9 (X&Y solutions Inc., Boston, MA) and R software, version 3.2.0 (R Foundation for Statistical Computing, Vienna, Austria).

The median and 25th and 75th percentiles of food consumption and nutrient intake were calculated for the two FFQs and three 24-h recalls. Differences in intakes between the two administrations (FFQ1 and FFQ2) and between two methods (average of two FFQs and average of three 24-h recalls) were tested by using Wilcoxon signed rank test. Before further analysis, all dietary variables were log-transformed to have a normal distribution. In all analyses, statistical significance was set as *p* < 0.05.

Intraclass correlation coefficients were calculated to test the reproducibility between the two administrations of FFQ. Pearson correlations were estimated to validate the FFQ against 24-h recall. As most of the intakes of food and nutrient correlated with total energy intake, an energy adjustment method was performed by using the residual method of Willett, in which residuals were computed from a regression model [8]. To take into account the effect of random within-person error, the energy-adjusted Pearson correlation coefficient was de-attenuated according to the formula: R = r(1 + λ_x_/n_x_)^0.5^, in which λ_x_ was the ratio of the within- and between-person variances for *x*, and n_x_ was the number of replicates for the x variable [8]. For this study, n was 3.

A quintile classification analysis was used to category the food and nutrient intake values calculated by the FFQ and the three 24-h recalls. The percentage of correctly classified subjects into the same or same and adjacent or extreme quintiles was calculated. Additionally, Bland–Altman plots were performed to assess the agreement between the FFQ and three 24-h recalls for all nutrients and food groups [21].

## Results

### The characteristics of the participants

The baseline characteristics of the participants were shown in Table 1. They had a mean age of 28.1 years. Based on the value of pre-pregnancy BMI, 58.5 % of participants had a normal weight (BMI = 18.5-23.9 kg/m^2^) prior to becoming pregnant, 28.5 % underweight, 9.8 % overweight and 3.2 % obese. The mean weekly weight gain was 0.42 kg between the first and second FFQ administrations. More than half of the women had educated for at least 16 years. Most pregnant women (87.8 %) were nulliparous. Among the participants, about 75 % had a personal income higher than 3000 yuan (about 480 US Dollars) each month. Most of participants (93.5 %) took supplement and 65 % of women had nausea or vomiting during their first trimester. A small number of participants reported having ever consumed alcohol (4.1 %), smoked or passively smoked (9.8 %) during their first trimester.

**Table 1:** Characteristics of 123 pregnant Chinese women in the study, January 2013 to April 2014

Characteristics	Subjects
Age (years), mean (SD)	28.1(3.7)
Weight (kg), mean (SD)	55.0(9.5)
Height (m), mean (SD)	1.61(0.49)
Weekly weight gain between two FFQs(kg), mean (SD)	0.42(0.37)
Age (years), n(%)	
≤25	31(25.2)
26-30	67(54.5)
≥31	25(20.3)
Pre-pregnancy BMI ( kg/m^2^), n(%)	
<18.5	35(28.5)
18.5-23.9	72(58.5)
24.0-27.9	12(9.8)
≥28	4(3.2)
Education (schooling years), n(%)	
≤9	8(6.5)
10-12	22(17.9)
13-15	38(30.9)
≥16	52(42.3)
Missing values	3(2.4)
Parity, n(%)	
0	108(87.8)
≥1	15(12.2)
Average personal income (yuan/ month, 1 yuan = 0.16 US Dollars), n (%)	
≤1000	2(1.6)
1001-2999	29(23.6)
3000-4999	54(43.9)
5000-9999	34(27.6)
≥10000	4(3.3)
Health status or lifestyle variables during first trimester, n (%)	
The presence of nausea or vomiting	80(65.0)
Use of supplements	115(93.5)
Drinking	5(4.1)
Smoking or passive smoking	12(9.8)

### Reproducibility

As shown in Table 2, the median intakes of all food groups and nutrients, except for beans, fruits, nuts, beverages, vitamin C, vitamin E and iodine, were generally higher when estimated by FFQ2 than by FFQ1. The most significant differences between the first and the second FFQ among food groups were for fish, eggs and milk and milk products, and among nutrients, for protein, high-quality protein, cholesterol, choline, zinc and selenium. The intraclass correlation coefficients for food groups ranged from 0.23 for nuts to 0.49 for fruits; and for nutrients ranged from 0.24 for iodine to 0.58 for selenium and all correlations being statistically significant (*p* < 0.01 except for iodine *p* = 0.01). The average of correlation coefficients was 0.32 for food groups and 0.44 for nutrients.

**Table 2:** Reproducibility study: median daily intakes of foods and nutrients estimated by FFQs and intraclass correlation coefficients between FFQ1 and FFQ2 completed by 123 pregnant Chinese women, January 2013 to April 2014

Food groups or nutrients	FFQ1	FFQ2	%FFQ2/FFQ1	Intraclass correlation coefficients(95 % CI)^†^	*P* Value^△^
	Median (P_25_,P_75_)	Median(P_25_,P_75_)	Median		
Food groups					
Cereals (g/day)	175 (135-220)	187(160-225)	107	0.25(0.08,0.41)	<0.01
Meats (g/day)	27(7-46)	29(13-54)	107	0.37(0.20,0.51)	<0.01
Fish (g/day)	19(7-43)	25(11-47) ^*^	132	0.40(0.24,0.54)	<0.01
Eggs (g/day)	43 (21-50)	50(29-50)^*^	116	0.30(0.13,0.45)	<0.01
Beans (g/day)	7 (3-15)	6(3-15)	86	0.26(0.09,0.42)	<0.01
Vegetables (g/day)	255(165-394)	283(183-454)	111	0.34(0.18,0.49)	<0.01
Fruits (g/day)	428 (287-566)	421(278-574)	98	0.49(0.34,0.61)	<0.01
Nuts (g/day)	11(4-20)	10(3-20)	91	0.23(0.05,0.39)	<0.01
Milk & milk products (g/day)	143 (32-257)	200(46-305) ^*^	140	0.31(0.14,0.46)	<0.01
Beverages (ml/day)	16(0-96)	0(0-57)	0	0.24(0.06,0.40)	<0.01
Nutrients					
Energy (kcal/day)	1713.0 (1370.0-2038.0)	1776.0(1510.0-2140.0)	104	0.52(0.37,0.63)	<0.01
Protein (g/day)	52.2 (41.3-67.3)	58.9 (46.4-72.9) ^*^	113	0.52(0.38,0.64)	<0.01
High quality protein (g/day)	22.7(14.0-33.2)	27.7 (20.9-37.6) ^*^	122	0.48(0.33,0.60)	<0.01
Fat (g/day)	50.5 (42.0-58.5)	53.2 (45.4-61.6)	105	0.44(0.29,0.57)	<0.01
Carbohydrate (g/day)	262.1(200.1-314.9)	267.7 (215.5-313.0)	102	0.43(0.28,0.56)	<0.01
Dietary fiber (g/day)	14.1 (10.4-19.3)	14.8 (11.0-21.3)	105	0.37(0.21,0.52)	<0.01
Cholesterol (mg/day)	335.4 (196.6-407.1)	373.6 (284.4-451.6) ^**^	111	0.35(0.18,0.49)	<0.01
Vitamin A (μg/day)	853.0 (566.3-1200.3)	884.7(615.6-1259.9)	104	0.41(0.26,0.53)	<0.01
Thiamin (mg/day)	1.0 (0.8-1.3)	1.0 (0.8-1.2)	100	0.44(0.28,0.57)	<0.01
Riboflavin (mg/day)	1.0 (0.8-1.5)	1.2 (0.9-1.7)	120	0.51(0.37,0.61)	<0.01
Vitamin C (mg/day)	160.2 (112.8-213.3)	153.0 (115.5-199.7)	96	0.40(0.24,0.54)	<0.01
Vitamin E (mg/day)	7.9 (5.4-10.2)	7.8 (5.9-9.9)	99	0.34(0.18,0.49)	<0.01
Choline (mg/day)	83.1 (53.7-104.4)	95.6 (71.6-113.1) ^*^	115	0.31(0.15,0.46)	<0.01
Niacin (mg/day)	11.3 (8.1-13.7)	11.8 (9.5-15.5)	104	0.47(0.32,0.60)	<0.01
Folate (μg/day)	122.4 (88.6-168.7)	124.1 (91.4-201.4)	101	0.38(0.22,0.52)	<0.01
Calcium (mg/day)	553.1 (392.3-857.4)	639.8 (460.8-909.0)	116	0.44(0.29,0.58)	<0.01
Phosphorus (mg/day)	901.7 (706.7-1138.0)	988.2 (794.1-1246.4)	110	0.56(0.43,0.67)	<0.01
Potassium (mg/day)	2259.7(1736.2-3134.5)	2404.2(1934.7-3159.2)	106	0.53(0.39,0.65)	<0.01
Sodium (mg/day)	450.2 (269.0-744.7)	463.0 (320.6-825.2)	103	0.50(0.35,0.62)	<0.01
Magnesium (mg/day)	285.0 (216.7-397.1)	303.4 (236.3-396.6)	106	0.53(0.39,0.65)	<0.01
Iron (mg/day)	18.1 (13.8-23.6)	20.2 (15.2-25.3)	111	0.40(0.24,0.54)	<0.01
Zinc (mg/day)	9.1 (7.0-10.9)	9.9 (7.9-12.4) ^*^	109	0.47(0.32,0.60)	<0.01
Selenium (μg/day)	32.8 (24.0-42.9)	36.2 (27.1-48.6) ^**^	110	0.58(0.44,0.68)	<0.01
Copper (mg/day)	2.0 (1.4-2.5)	2.1 (1.5-2.6)	105	0.40(0.24,0.54)	<0.01
Iodine (μg/day)	15.9 (7.7-24.5)	14.9 (8.4-24.1)	94	0.24(0.06,0.40)	0.01

^†^ Based on log-transformed values

*p<0.05, **p<0.01 Compared with intakes estimated by FFQ1 using Wilcoxon’s signed rank test

^△^*P* Value of intraclass correlation coefficients between two FFQ administrations

### Validity

On the whole, the median daily intakes of food groups and nutrients assessed by the average of two FFQs tended to show higher values than those from the average of three 24-h recalls, except for cereals, meats, nuts, beverages, high quality protein, fat, sodium and selenium (Table 3). The unadjusted Pearson correlation coefficients for foods ranged from 0.28 for beans to 0.53 for fruits. For nutrients, the unadjusted Pearson correlation coefficients ranged from 0.15 for iodine to 0.59 for protein. Pearson correlation coefficients decreased after energy adjustment for most food groups and nutrients except eggs and choline which increased slightly. The most obvious changes compared with the unadjusted values occurred in phosphorus (from 0.54 to 0.19) and riboflavin (from 0.34 to 0.11). The energy-adjusted coefficients for foods or nutrients were statistically significant (*p* < 0.01 or *p* < 0.05) with the exception of riboflavin, iodine and sodium (p > 0.05). After de-attenuation, the average of the correlation coefficients (0.43) was improved relative to the unadjusted coefficients (0.40). Considering the effect of attenuation, the coefficients ranged from 0.11 for iodine to 0.63 for protein.

**Table 3:** Validation study: median daily intakes of food and nutrients based on the average of two FFQs and the average of three 24-h recalls; Pearson correlation coefficients between FFQs and 24-h recalls completed by 123 pregnant Chinese women, January 2013 to April 2014

Food groups or nutrients	Average of two FFQ Median (P_25_,P_75_)	Average of three 24HR Median (P_25_,P_75_)	%FFQ/24HR (Median)	Pearson correlation coefficients^†^			
				Un-adjusted	Energy adjusted	Energy adjusted and de-attenuated	*P* Value^△^
Food groups							
Cereals (g/day)	186(158 -212)	200(150-240)^#^	93	0.36	0.32	0.37	<0.01
Meats (g/day)	31(14-50)	48(18-78)^##^	65	0.47	0.31	0.41	<0.01
Fish (g/day)	25(13-43)	25(0-64)	100	0.42	0.27	0.38	<0.01
Eggs (g/day)	39(29-50)	30(20-53)	130	0.35	0.36	0.41	<0.01
Beans (g/day)	8(4-15)	5(0-13)^##^	160	0.28	0.30	0.35	<0.01
Vegetables (g/day)	289 (192-423)	275(175-345)^#^	105	0.35	0.33	0.39	<0.01
Fruits (g/day)	420(295-560)	300(200-433)^##^	140	0.53	0.48	0.56	<0.01
Nuts (g/day)	5(12-20)	9(0-25)	56	0.45	0.37	0.56	<0.01
Milk & milk products (g/day)	170(76-280)	125 (0-240)^##^	136	0.45	0.43	0.50	<0.01
Beverages (ml/day)	27(0-86)	0(0-0)^##^	---	0.30	0.30	0.38	<0.01
Nutrients							
Energy (kca/dayl)	1754.5 (1479.5-2013.0)	1705.5 (1446.5-1999.0)	103	0.54			
Protein (g/day)	56.7 (45.2-69.3)	56.8 (47.2-71.2)	100	0.59	0.55	0.63	<0.01
High quality protein (g/day)	26.1 (18.4-34.9)	27.5 (20.1-38.3)	95	0.58	0.48	0.54	<0.01
Fat (g/day)	51.9 (45.2-59.3)	52.8 (42.2-69.0)^#^	98	0.50	0.39	0.46	<0.01
Carbohydrate (g/day)	263.9 (221.0-301.2)	243.1 (209.4-284.6)^#^	109	0.45	0.36	0.42	<0.01
Dietary fibe (g/day)	15.3 (11.6-20.8)	12.4 (10.4-15.6)^##^	123	0.35	0.26	0.31	<0.01
Cholesterol (mg/day)	347.3 (268.1-427.3)	319.8 (207.4-485.2)	109	0.51	0.46	0.54	<0.01
Vitamin A (μg/day)	872.0(655.8.0-1146.0)	556.3 (373.9-746.5)^##^	157	0.38	0.18	0.21	0.05
Thiamin (mg/day)	1.0 (0.9-1.2)	0.9 (0.7-1.1)^##^	111	0.27	0.21	0.26	0.02
Riboflavin (mg/day)	1.1 (0.9-1.7)	0.9 (0.7-1.1)^##^	122	0.36	0.11	0.13	0.21
Vitamin C (mg/day)	164.4 (125.3-204.1)	123.8 (92.6-178.0)^##^	133	0.40	0.34	0.39	<0.01
Vitamin E (mg/day)	8.0 (6.1-9.7)	7.2 (5.1-9.2)^#^	111	0.35	0.28	0.31	<0.01
Choline (mg/day)	89.0 (68.9-102.8)	75.3 (52.3-104.8)^#^	118	0.39	0.40	0.47	<0.01
Niacin (mg/day)	11.8 (9.6-14.5)	11.8 (9.2-14.9)	100	0.54	0.43	0.50	<0.01
Folate (μg/day)	133.6 (94.0-170.1)	98.5 (64.0-144.5)^##^	136	0.38	0.27	0.32	<0.01
Calcium (mg/day)	608.4 (472.0-860.9)	503.1 (407.1-643.2)^##^	121	0.46	0.37	0.42	<0.01
Phosphorus (mg/day)	941.5 (748.1-1183.8)	930.0 (738.8-1128.2)	101	0.54	0.19	0.22	0.04
Potassium (mg/day)	2481.5 (1799.2-3058.8)	2041.0 (1641.6-2466.5)^##^	122	0.49	0.38	0.43	<0.01
Sodium (mg/day)	481.0 (312.0-735.0)	600.3 (359.7-892.6)	80	0.20	0.11	0.13	0.22
Magnesium (mg/day)	297.9 (233.8-380.1)	274.9 (216.1-347.6)^##^	108	0.49	0.35	0.41	<0.01
Iron (mg/day)	19.6 (15.3-26.2)	17.8 (14.2-21.1)^##^	110	0.34	0.21	0.23	0.02
Zinc (mg/day)	9.5 (7.8-11.5)	9.4 (7.6-11.6)	101	0.33	0.19	0.23	0.03
Selenium (μg/day)	35.4 (27.6-44.5)	35.9 (28.3-54.8)^#^	99	0.58	0.51	0.61	<0.01
Copper (mg/day)	2.1 (1.7-2.6)	2.0 (1.6-2.5)	105	0.31	0.21	0.24	0.02
Iodine (μg/day)	15.4 (10.8-23.0)	9.1 (5.0-17.5)^##^	169	0.15	0.10	0.11	0.30

^†^ Based on log-transformed values; ^#^*p*<0.05, ^##^*p*<0.01 Compared with average intakes estimated by two FFQ administrations using Wilcoxon’s signed rank test

^△^*P* Value of energy adjusted Pearson correlation coefficients between average intakes obtained from two FFQs and three 24-h recalls

Table 4 presented the percentage of participants categorized into quintiles by food consumption and nutrient intake (unadjusted estimates) obtained from FFQs and 24-h recalls. A high proportion of study participants (>70 %) were categorized into the same or adjacent quartiles for estimated food consumption such as meats, fish, fruits, milk and milk products, and for estimated nutrients intake such as protein, high-quality protein, fat, thiamin, riboflavin, niacin, phosphorus, potassium, magnesium and selenium. On average, 67.0 % (51.2 %-80.5 %) of women were classified into the same or adjacent quintiles based on their food intakes, while 68.5 % (56.1 %-77.2 %) of women were classified as such based on nutrient intakes. Extreme misclassifications were very low for both foods (average of 2.0 %) and nutrients (average of 2.2 %).

**Table 4:** Validation study: cross-classification of intakes of food and nutrients based on the average of two FFQs and the average of three 24-h recalls completed by 123 pregnant Chinese women, January 2013 to April 2014

Food groups or nutrients	Same quintile (%)	Same or adjacent quintile (%)	Extreme quintile (%)
Food groups			
Cereals	27.6	56.1	4.1
Meats	32.5	74.8	1.6
Fish	29.3	72.4	1.6
Eggs	32.5	68.3	1.6
Beans	22.8	60.2	0.8
Vegetables	27.6	66.7	4.1
Fruits	30.9	71.5	0.0
Nuts	35.0	68.3	3.3
Milk & milk products	40.7	80.5	0.0
Beverages	23.6	51.2	3.3
Nutrients			
Energy	32.5	69.9	1.6
Protein	39.0	74.8	1.6
High quality protein	35.8	70.7	1.6
Fat	41.5	70.7	2.4
Carbohydrate	26.8	69.1	2.4
Dietary fiber	33.3	67.5	1.6
Cholesterol	35.8	69.9	0.8
Vitamin A	34.1	66.7	1.6
Thiamin	33.3	74.0	2.4
Riboflavin	36.6	73.2	2.4
Vitamin C	28.5	59.3	0.8
Vitamin E	26.8	67.5	1.6
Choline	30.1	64.2	2.4
Niacin	29.3	74.0	0.8
Folate	29.3	69.9	3.3
Calcium	35.8	63.4	2.4
Phosphorus	31.7	72.4	3.3
Potassium	27.6	74.8	3.3
Sodium	22.8	58.5	4.1
Magnesium	31.7	71.5	2.4
Iron	26.8	66.7	3.3
Zinc	25.2	66.7	2.4
Selenium	32.5	77.2	0.0
Copper	29.3	63.4	0.0
Iodine	22.0	56.1	7.3

**Fig. 2:**
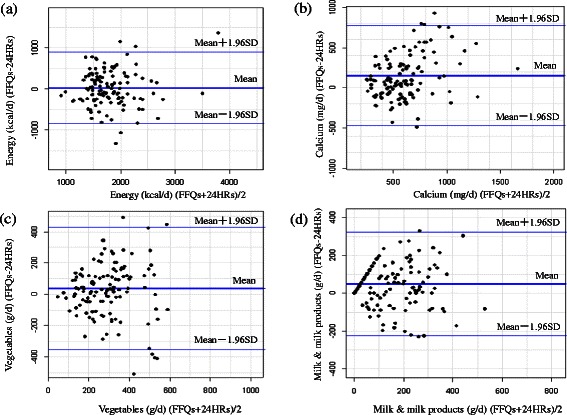
Bland–Altman plot showing agreement between the average of food frequency questionnaires (FFQs) and the three 24-h recalls (24HRs) in estimating the intakes of (**a**) energy, (**b**) calcium, (**c**) vegetables and (**d**) milk & milk products. (SD - standard deviation)

The results of Bland–Altman plots were shown in Fig. 2 for energy, calcium, vegetables and milk and milk products. According to Fig. 2, most of the points fell within the 95 % limits of agreement (LOAs) and no linear trend showed between the differences and means for nutrients and food groups. The resulting plots produced for all other nutrients and food groups were similar to those in Fig. 2 (data not shown).

## Discussion

In order to identify the associations between maternal diet during pregnancy and health outcomes of offspring, a semi-quantitative FFQ consisting of 61 food items was designed to evaluate intakes of foods and nutrients of urban pregnant women from central China. Participants at 12–13 weeks of gestation were recruited at their first prenatal care visit. Dietary intake of pregnant women may change considerably during pregnancy due to appetite fluctuations caused by nausea or vomiting, food preferences and increased energy requirements. Thus, intake in a specific period of pregnancy may not be representative of the whole gestation. However, several studies showed that a single FFQ was able to capture dietary intake throughout the whole pregnancy [22, 23]. In the present study, we evaluated the reproducibility and relative validity of the FFQ in early second trimester (12 ~ 16 weeks of gestation), by which nausea or vomiting usually subsided [24].

In the reproducibility study, the intraclass correlation coefficients ranged from 0.23 (nuts) to 0.49 (fruits) for foods and from 0.24 (iodine) to 0.58 (selenium) for nutrients. Erkkola reported slightly higher coefficients than those of the present study for both food groups and nutrients intakes assessed by a self-administered 181-item FFQ designed for Finnish pregnant women [25]. The complexity of cooking methods for Chinese foods may be responsible for the low correlation coefficients of FFQ used for Chinese population because many food items mix each other in Chinese dishes make it difficult to estimate the accurate amount of each food item. However, most of the correlation coefficients for nutrients showed in our study were similar or comparable to those showed in other reproducibility studies in Chinese population or pregnant women of other countries [15, 26, 27]. We found that the coefficient of fruits was higher than that of any other foods, as seen in some earlier studies [28, 29]. The main reason might be that fruits were the preference for most of the pregnant women and consumed regularly and separately from a Chinese dish, while other foods such as meats, eggs or beans were usually consumed as a part of mix dishes, leading to the difficulty being estimated. Several previous studies suggested that the FFQ was not effective at estimating fruits or vegetables consumption for pregnant women [25, 30], because the consumption of these foods assumed to increase with the progress of pregnancy and compromised the results of correlation. However, the correlations for vegetables or fruits consumption between two FFQs in the present study were good although the vegetables intake level derived from FFQ2 was a little higher than that derived from FFQ1. In Chinese traditional belief, animal foods but not vegetables or fruits should be highly recommended in the diet for pregnant women. In fact, the intakes of several animal foods such as fish, eggs and milk and milk products, and intakes of several nutrients abundant in these foods such as high-quality protein, cholesterol, choline and selenium were significantly higher in the FFQ2 than those in the FFQ1, which would reflect the real dietary changes as the pregnancy progresses rather than measurement errors. These results were consistent with the previous study that evaluated the FFQ in Chinese pregnant women [14, 15].

To validate FFQ, several commonly used methods were available, including diet records, weighed diet records and 24-h recalls [8, 23, 31]. In the present study, a three day 24-h recall method was used as the reference standard. The average of unadjusted correlation coefficients between FFQs and 24-h recalls in our study were 0.41 for food groups (0.28-0.53) and 0.40 for nutrients (0.15-0.59). The energy adjustment method and correction for attenuation were used to eliminate the effects of differences in the total energy intake between subjects and random within-person variation [8]. Energy-adjustment led to the decrease of correlation for almost all food groups and nutrients, which might be due to the high between-person variation of intake in our study subjects. After energy adjustment and de-attenuated correction, the mean coefficient values of foods increased from 0.41 to 0.45 (0.35-0.56), while those of nutrients decreased from 0.40 to 0.36 (0.11-0.63). These correlations showed in the present study were higher than or comparable to the published results in other validation studies among pregnant women [9, 14, 32, 33]. Li et al. [14] reported the correlation coefficients ranged from 0.12 to 0.54 for food groups and from 0.19 to 0.55 for nutrients in pregnant women from rural area of China. In a study conducted among Brazilian pregnant women, the average of the de-attenuation correlation coefficients for nutrients was 0.35 (from −0.1 to 0.6) [33].

In most validation studies, correlation coefficients between instruments ranged from 0.30 to 0.49 are considered reasonable and more than 0.5 are good [34]. In the present study, correlation coefficients for all food groups, macronutrients and most of micronutrients were significantly good or acceptable but for some micronutrients, especially iodine, sodium and riboflavin were poor. Although the correlations for several other micronutrients (such as vitamin A, thiamin, phosphorus, iron, zinc and copper) were significant, but also considered as not good, being lower than 0.3. The good source of iodine and sodium in Chinese diets is iodized salt. One possible reason of the poor correlation was the inaccurate estimation of iodized salt intake by our FFQ in this population. The subjects just were asked to select their taste as “light”, “medium” or “strong” instead of reporting the frequency or amount of iodized salt used in the diet. The low correlation of other micronutrients (such as vitamin A, thiamin, riboflavin iron and zinc) could be partly explained by low frequency consumption of food enriched these nutrients in Chinese population.

Except for correlation coefficients, another more appropriate way to access the agreement between two methods is by cross-classification and the percentage of agreement [35]. The mean percentages of participants classified into the same or adjacent quintile observed in the present study were 67.0 % for food and 68.5 % for nutrients, while misclassified into extreme quintile were only 2.0 % for food and 2.2 % for nutrients. This level of agreement was similar or comparable to that reported in other validation studies in pregnant women [15, 23, 25]. Bland–Altman method was also used to graphically assess agreement between the two methods. The distribution of the points within the LOAs indicated that the two methods were comparable, although the mean of the differences suggested that the FFQ slightly overestimated energy, calcium, vegetables and milk and milk products. The agreement between the two methods was decreased with the increase in dietary intake which might provide evidence that women who had higher consumption tend to over-report or under-report their consumption by FFQs, as seen in several previous validation studies among pregnant women [9, 36].

In the present study, the FFQ seemed to slightly overestimated intakes of most foods and nutrients compared with the 24-h recalls. Most of the previous validation studies among pregnant women had reported similar overestimates using FFQs compared with food records or 24-h recalls [25, 36, 37]. Overestimation may reflect over-reporting of the frequency of consumption of foods, larger portion size estimation by the FFQ or under-reporting of consumption by the food records or 24-h recalls. Since FFQs target ranking individuals by intake of specific food groups or nutrients rather than providing absolute values of intakes, overestimation is not a problem in epidemiologic studies if the classification of individuals by food groups or nutrients intake levels is acceptable [23, 25, 30].

There were limitations in the present study. The main limitation was the fact that FFQ and 24-h recall had similar sources of errors such as recall bias. However, given the actual situation and compliance of pregnant women, 24-h recall was considered more appropriate than diet records or weighed records in the present study. Another limitation was the use of only a small number of 24-h recalls as the standard for estimating the long-term dietary intake of pregnant women. Due to the high day-to-day variations of dietary intake in pregnant women, limited number of recalls might under-report or over-report usual food intake and thus increased the disagreement between the methods. Additionally, the correlations between two FFQs would have been compromised by the variations of dietary intake as pregnancy progresses due to an interval of 3 ~ 4 weeks between two FFQ administrations in the present study.

## Conclusions

In summary, our FFQ exhibited acceptable reproducibility and reasonable validity in assessing most food and nutrient intakes among pregnant women. Based on the present study, this FFQ appears to be appropriate for investigating the relationship between dietary intakes of pregnant women and related health outcomes in central urban China. However, the FFQ should be evaluated in future studies in other urban regions of China.

## Abbreviations

FFQ: Food frequency questionnaire
24 HR: 24-h recall
TMCHC: Tongji Maternal and Child Health Cohort
BMI: Body mass index
LOAs: Limits of agreement
